# Changes in circulating procalcitonin versus C-reactive protein in predicting evolution of infectious disease in febrile, critically ill patients

**DOI:** 10.1186/cc11968

**Published:** 2013-03-19

**Authors:** S Hoeboer, J Groeneveld

**Affiliations:** 1 VU University Medical Center, Amsterdam, the Netherlands; 2 Erasmus Medical Center, Rotterdam, the Netherlands

## Introduction

Although absolute values for C-reactive protein (CRP) and procalcitonin (PCT) are well known to predict sepsis in the critically ill, it remains unclear if and how changes in CRP and PCT predict evolution of infectious disease and how they compare in this respect.

## Methods

In 72 critically ill patients with new-onset fever, CRP and PCT were measured on day 0, 1, 2 and 7 after inclusion, and their clinical course was documented over 1 week with follow-up to day 28. Infection was microbiologically defined, as was bloodstream infection; septic shock was defined as infection plus shock.

## Results

From peak at day 0 to 2 to day 7, CRP decreases most when (bloodstream) infection and septic shock (day 0 to 2) resolve and increases most when complications such as a new (bloodstream) infection or septic shock (day 3 to 7) supervene (area under the receiver operating characteristic curve 0.70 or higher, *P *= 0.04 or lower). PCT decreases most when septic shock resolves (AUC 0.72, *P *= 0.007) and increases most when a new bloodstream infection or septic shock supervenes (AUC 0.82 or higher, *P <*0.001). The day 7 value of PCT rather than of CRP was predictive for 28-day outcome (AUC 0.70, *P *= 0.005).

## Conclusion

The data, obtained during ICU-acquired fever and infections, suggest that CRP and PCT changes predict the course of infectious disease and its complications. CRP may be favoured over PCT courses in decisions on appropriateness and duration of antibiotic treatment, whereas PCT rather than CRP courses may help predicting complications such as bloodstream infection, septic shock and mortality.

